# Microbiota influence the development of the brain and behaviors in C57BL/6J mice

**DOI:** 10.1371/journal.pone.0201829

**Published:** 2018-08-03

**Authors:** Jing Lu, Sylvia Synowiec, Lei Lu, Yueyue Yu, Talitha Bretherick, Silvia Takada, Vasily Yarnykh, Jack Caplan, Michael Caplan, Erika C. Claud, Alexander Drobyshevsky

**Affiliations:** 1 Department of Pediatrics, Neonatology, Pritzker School of Medicine, the University of Chicago, Chicago, Illinois, United States of America; 2 Department of Pediatrics, NorthShore University HealthSystem Research Institute, Evanston, Illinois, United States of America; 3 Laboratório de Neurogenética, Federal University of São Paulo, São Paulo, Brazil; 4 Department of Radiology, University of Washington, Seattle, Washington, United States of America; 5 Research Institute of Biology and Biophysics, Tomsk State University, Tomsk, Russian Federation; 6 Department of Chemical Engineering, University of Illinois at Urbana-Champaign, Urbana-Champaign, Illinois, United States of America; Biomedical Sciences Research Center Alexander Fleming, GREECE

## Abstract

We investigated the contributions of commensal bacteria to brain structural maturation by magnetic resonance imaging and behavioral tests in four and 12 weeks old C57BL/6J specific pathogen free (SPF) and germ free (GF) mice. SPF mice had increased volumes and fractional anisotropy in major gray and white matter areas and higher levels of myelination in total brain, major white and grey matter structures at either four or 12 weeks of age, demonstrating better brain maturation and organization. In open field test, SPF mice had better mobility and were less anxious than GF at four weeks. In Morris water maze, SPF mice demonstrated better spatial and learning memory than GF mice at 12 weeks. In fear conditioning, SPF mice had better contextual memory than GF mice at 12 weeks. In three chamber social test, SPF mice demonstrated better social novelty than GF mice at 12 weeks. Our data demonstrate numerous significant differences in morphological brain organization and behaviors between SPF and GF mice. This suggests that commensal bacteria are necessary for normal morphological development and maturation in the grey and white matter of the brain regions with implications for behavioral outcomes such as locomotion and cognitive functions.

## Introduction

Microbial communities in the infant intestine, or the intestinal microbiota, are increasingly considered as a modifiable factor to influence the development of brain and host behavior [1–4]. Microbiota is acquired around birth and develops to a relative stable community contemporaneously with nervous system development during the first 2–3 years and may have direct and profound impacts on cognition and behavior later in life [5]. Gut microbiota has been shown to be involved in the early programming of brain circuits that mediate stress response, motor activity, anxiety-like behavior and cognitive functions in early childhood as well as the potential pathogenesis of neurodevelopmental disorders, such as autism, attention-deficit/hyperactivity disorder, and schizophrenia [6–11].

During the first two years of postnatal life, profound changes occur in the nervous system: a massive outgrowth of dendrites and axons, synaptogenesis, expansion of glia cells and myelination [12, 13]. Quantitative magnetic resonance imaging (MRI)-based volumetric studies have consistently shown that brain morphology changes dynamically from birth to adolescence into adulthood [14, 15]. Studies have demonstrated a general positive correlation between grey matter volume and IQ performance in normal children and adolescents from five to 17 years old [16] as well as in adults[17]. Furthermore, age-related expansion and myelination of the white matter are prominent indictors of brain maturation and cognitive function later in life [18].

The benefits of the commensal microbiota on host physiology and on brain function and development are being explored using germ free (GF) mice [19], demonstrating the importance of microbiota in regulating multiple brain developmental processes including synaptogenesis and related second messenger pathways [6, 20], myelination [21], the hippocampal serotonergic system [7], blood-brain barrier permeability[22] as well as the hypothalamic–pituitary–adrenal axis response to stress [9], and exploratory and anxiety-like behaviors [23]. Studies using stereological volumetric estimation have revealed regional volumetric changes such as enlarged amygdala and hippocampus, attributed to the reduction in dendritic branching in adult germ free mice [24]. However, associations between brain structure and microbiota profiles have not been described in mice using MRI neuroimaging techniques. Application of non-invasive and high throughput MRI methods would be of great benefit to study developmental effects of microbiota on brain structure, as changes are often subtle, distributed across the brain and evolve with time. Furthermore, the effect of microbiota on the brain is likely to involve multiple regions and cellular targets, but most of the current studies are focused on one or two specific regions and behaviors. In this study we specifically employed unbiased quantitative neuroimaging methods across the whole brain in order to assess microstructural and volumetric differences between mice with commensal bacteria also known as specific pathogen free (SPF) and GF mice at four weeks of age (considered as juvenile) and 12 weeks of age (considered as adult).

In light of intriguing reports of myelination disturbances in the prefrontal cortex of GF mice [21] we placed a special emphasis on assessing myelination and white matter development at juvenile and adult age. Myelination ensures efficient transfer of information between neural regions postnatally and is considered a crucial indicator of brain maturation [18]. A novel *in vivo* quantitative MRI method, macromolecular proton fraction (MPF) mapping, was employed to reconstruct parametric maps of a relative amount of macromolecular protons causing the magnetization transfer effect which provides a biomarker of myelination in neural tissues [25, 26]. The method has been validated with histological markers of myelin in the normal rat brain [27] and in a murine model of demyelination [28]. The method has high resolution, is fast and independent of directional organization of the tissue which uniquely provides opportunity to assess myelination in gray matter structures [28, 29] and provides complimentary information to diffusion tensor imaging in white matter that is also used in this study.

Understanding of how early life modifications in gut microbiota contribute to vulnerability to behavioral and cognitive disorders is of paramount importance in clinical and behavioral neuroscience. The aim of this study was to investigate the contributions of intestinal bacteria to behavior and related brain structures and function using the C57BL/6J strain of SPF and GF mice. Since both gender and age related dependent microbiota influences on the brain are expected [30, 31], systematic characterization of morphological and myelination changes across the whole brain between males and females in juvenile and adult mice (four and 12 weeks of age) was performed. Our data demonstrate that commensal bacteria are necessary for normal morphological development and maturation in the grey and white matter of the brain regions with implications for behavioral outcomes such as locomotion and cognitive functions.

## Materials and methods

### Animals and general plan of the experiments

The study received approval by the Institutional Animal Care and Use Committees of NorthShore University HealthSystem (NorthShore) under the protocol EH16-264 and the University of Chicago under the protocol No. 71703, and all studies were conducted strictly in accordance with the United States Public Health Service's Policy on Humane Care and Use of Laboratory Animals and approved Animal Care and Use at The University of Chicago. Germ free (GF) C57BL/6J mice, originally obtained from the Jackson Laboratory (Bar Harbor, Maine), were bred and maintained in the gnotobiotic facility of the Digestive Disease Research Core Center at the University of Chicago. The GF colony was routinely tested for microbes and parasites by the facility’s staff to ensure germ-free conditions. Specific pathogen free (SPF) C57BL/6J mice were obtained from the Jackson Laboratory. Mice were tested at two time points in development: four weeks (15 males and 15 females SPF, nine males and six females GF) and 12 weeks (13 males and 13 females SPF, seven males and seven females GF). Separate cohorts were used for each time point. There was no difference in body weight between the SPF and GF mice of the same gender (Table 1). Animals were transported to NorthShore in sterile containers 3–5 days before testing to allow an acclimation period. Upon arrival at NorthShore mice were housed in individually ventilated cages with HEPA filter until the behavioral testing began. Morris water maze was performed on days 1–5. On days 1–5, we began with Morris water maze at 10 am in the morning. Animals were allowed to recover for two hours before open field, elevated plus maze or social interaction test was performed in the afternoon on Day 1, 2, and 3, respectively. Fear conditioning was performed on days 7 and 8. After the behavioral testing, mice underwent volumetric and macromolecule proton fraction *in vivo* magnetic resonance imaging (MRI) on days 9 and 10. Immediately after *in vivo* MRI, mice were deeply anesthetized with sodium pentobarbital (100mg/kg) and transcardially perfused with 30 mL of phosphate-buffered saline (PBS) (pH 7.4) at room temperature (25°C). This was followed by infusion with 30 mL of 4% paraformaldehyde (PFA) in PBS with 2 mM ProHance (Bracco Diagnostics Inc., Princeton, NJ). Following perfusion, the heads were removed along with the skin, lower jaw, ears and the cartilaginous nose tip. The remaining skull structures containing the brain were allowed to postfix in 4% PFA at 4°C overnight. The samples were transferred and stored in a PBS and 2 mM ProHance solution and 0.01% sodium azide until they underwent *ex vivo* high resolution volumetric and diffusion weighted MRI. After imaging the brains were processed for immunohistochemistry.

**Table 1:** Body weights of experimental mice.

Age (week)	SPF (Body weight (g))		GF (Body weight (g))	
	Male	Female	Male	Female
**4**	15.21±0.50	14.61±0.27	17.20±0.71	13.46±0.47*
**12**	26.46±0.44	20.47±0.35*	26.05±0.65	21.16±0.30*

*Significantly smaller compared to respective male mice (*p*<0.001).

### Magnetic resonance imaging (MRI)

#### *In vivo* macromolecule proton fraction (MPF) imaging

**Fig 1:**
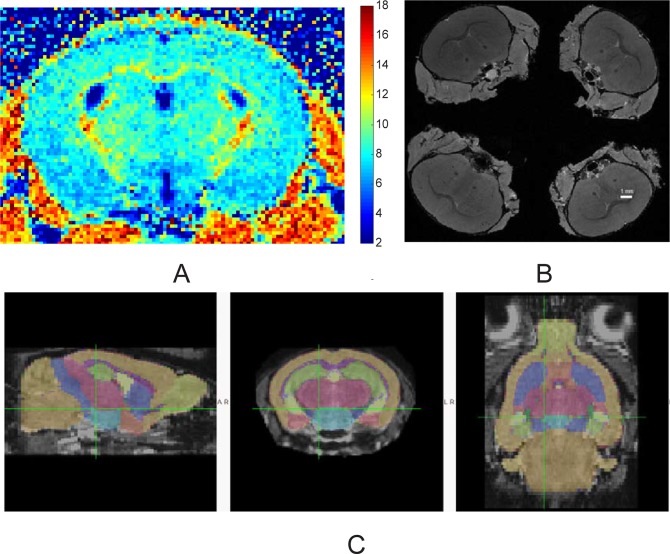
MRI methods. (A). Macromolecular proton fraction map of four weeks old mouse. Color bar indicates pseudo-color mapping of MPF in percent units. (B). High resolution T1-weighed volumes of *ex*-vivo mouse brain images with skulls in situ. A group of four brains were imaged at the same time. (C). Automatic structural parcellation of a mouse brain obtained from MT image using multi-atlas label fusion method.

Imaging was performed on a 14.1 T Bruker Avance imaging spectrometer (Bruker, Billerica, MA) using a 20-mm resonator. Mice were sedated with isoflurane (Abbot, IL) inhalation, diluted in air to 5% for induction and 1.5% for maintenance. Animal respiration rate and rectal temperature were monitored with a small animal physiological monitor (SAII's Small Animal Instruments, NY). Body temperature was maintained at 35^o^ C by maintaining in bore ambient temperature at 32 ^o^ C using the spectrometer gradient temperature controller. 3D MPF maps (Fig 1A) were obtained from three source images (Magnetization transfer (MT)-, Proton density (PD)-, and T1-weighted) using the single-point method with the synthetic reference image [32]. PD- and T1-weighted GRE images were acquired with TR/TE = 16/2.6 ms and α = 3° and 16°, respectively. MT-weighted images were acquired with TR/TE = 25/2.6 ms and α = 9°. Off-resonance saturation pulse was applied at the offset frequency 6 kHz with the effective saturation flip angle 500°. All images were acquired in the axial plane with whole-brain coverage and resolution 0.125x0.125x0.25 mm^3^. All images were obtained with four signal averages and the total scan time of 33 min. In all 3D imaging experiments, linear phase-encoding order with 100 dummy scans, slab-selective excitation, and fractional (75%) k-space acquisition in the slab selection direction were used. To correct for field heterogeneities, 3D B0 and B1 maps were acquired using the dual-TE (TR/TE1/TE2 = 20/2.9/5.8 ms, α = 8°) and actual flip-angle imaging (AFI) (TR1/TR2/TE = 13/65/4 ms, α = 60° methods, respectively [33]. All reconstruction procedures were performed using custom-written C-language software.

#### *Ex vivo* MRI

*Ex vivo* MR imaging occurred from seven to 21-day post-fixation. Skulls were immersed in non-aqueous media (fomblin Y, Sigma Aldrich, MO). Imaging was performed on a 14.1 T Bruker Avance imaging spectrometer (Bruker, Billerica, MA) using a 30-mm resonator. Four brains were imaged at the same time (Fig 1B). High resolution T1-weighted image for Tensor based morphometry was obtained with parameters TR/TE/NEX 17/4.8/8, with 60 μm isotropic resolution. Imaging time was four hours and ten minutes.

Diffusion tensor imaging (DTI) experiments consisted of 30 non-collinear directions diffusion weighted spin echo images with b = 0 and 1.0 ms/μm^2^, δ/Δ = 3/7 ms. Imaging parameters were TR/TE/NEX 7500/15.1/1, 40 axial slices 0.25 mm thick with no gap covering whole cerebrum. In-plane resolution was 125x125 μm and brain volumes were interpolated to isotropic 125 μm^3^. Imaging time was 11 hours 22 minutes. Diffusion tensor maps were calculated using multivariate linear fitting of signal attenuation from the acquired diffusion weighted images [34]. Fractional anisotropy (FA) maps were calculated [35] using in-house software written on Matlab (MathWorks, Natick, MA).

#### MRI data processing

Magnetization transfer images of mouse brains, obtained as a component of the *in vivo* MPF experiment and possessing excellent white/gray matter contrast, were used for automatic structural parcellation using the multi-atlas label fusion method, as detailed in [36]. Briefly, individual mouse head images were processed for brain extraction, intensity non-uniformity correction, affine registration to atlas images and label fusion. The publicly available MRMNeAt atlas database was used, containing 10 individually labeled C57BL/6J *in vivo* mouse brains. 21 white and gray matter structures from the atlas were labeled (Fig 1C) with the addition of prefrontal cortex ROI which was defined as cortex 2 mm from the beginning of cerebrum. Volumes of individual anatomical areas and values of MPF were compared between SPF and GF groups.

Tensor based morphometry analysis was performed on *ex vivo* high resolution T1-weighted images using FSL (http://www.fmrib.ox.ac.uk/fsl/) routines. Study specific templates were created for each studied age by iterative registration of skull-stripped (as above for *in-vivo* imaging) randomly selected five GF and five SPF brains. For each age, brains were affine-registered and a randomly selected brain of the corresponding age group concatenated and averaged. This averaged image was then flipped along the x-axis and the two mirror images re-averaged to obtain a first-pass, study-specific "affine" template. Second, the brains were re-registered to this "affine" template using non-linear registration, averaged, and flipped along the x-axis. Both mirror images were then averaged to create the final symmetric, study-specific "non-linear" template. All brains (five males and five females of SPF and GF mice for four and 12 week groups) were non-linearly registered to the study/age-specific template. For each voxel, the natural logarithm of the Jacobian determinant (JD) of the warp field was calculated and volumes were smoothed with Gaussian kernels, sigma = 0.1 mm. JD is a measure of the deformation of each voxel with respect to the atlas image. It can be thought of as the amount by which the volume of that voxel had to be multiplied to reach the consensus average. JD > 1 signifies expansion and <1 denotes shrinkage of that voxel volume with respect to the volume of the same voxel in the atlas image. Permutation-based non-parametric inference between SPF and GF groups was performed on JD using *randomize* FSL routine and threshold-free cluster enhancement options to control family-wise error rate.

For the analysis of *ex vivo* DTI data, a cross-subject voxel-wise Tract Based Spatial Statistics analysis (TBSS) [37, 38], was utilized as implemented in the FSL software (http://www.fmrib.ox.ac.uk/fsl/). All individual FA volumes were registered to a template, the mean FA-map was calculated and thinned to represent the mean FA skeleton. For each subject, voxel data were projected from FA maps to the nearest voxels on the mean FA skeleton. The skeleton voxel values were assigned to the maximum of the projected voxels. The values of voxels on the common skeleton were analyzed with voxel-wise cross-subject statistical analysis utilizing a general linear model [37, 38]. In the case of two groups, as in this study, testing the contrast between the group predictors is equivalent to an unpaired t-test of the mean difference between the groups. As a result of the procedure, statistical parametric maps were created containing *p*-values for the voxel-wise two-sample unpaired t-tests. The results were corrected for multiple comparisons by controlling the family-wise error rate. Threshold-Free Cluster Enhancement and 3000 permutations were used for nonparametric permutation inference FSL routine.

### Myelin staining

Myelin contents estimation was performed on one of the 20-micron thick series using Luxol Fast Blue (LFB) stain (Sigma-Aldrich, St Louis, MO). Brains were extracted from skulls, cryoprotected in 30% sucrose and frozen on dry ice. Serial 20 micron thick sections were cut 400 microns apart on a cryostat and mounted onto poly-L-lysine-coated slides (Sigma-Aldrich). All slides, consisting of five brains per group (males and females, SPF and GF, 40 slides total) were processed as one batch with identical settings. Sections were incubated in 0.1% LFB solution for two hours at 60°C, differentiated by dipping in 0.05% lithium carbonate solution for 20 seconds, with continuing differentiation by repeatedly dipping in 70% alcohol until gray-matter contrast developed. The sections were rinsed, dehydrated in alcohols, cleared in xylene and mounted. Each whole-brain LFB stained series was photographed using a digital camera with eight megapixel resolution using identical imaging parameters. Images were analyzed with ImageJ software (National Institutes of Health, Bethesda, MD). Intensities of gray scaled images were measured in several gray and white matter regions of standard size and standard location: 1.7 bregma–for frontal cortex, 0 bregma—for anterior commissure, striatum, -1.28 bregma for fimbria, corpus callosum, internal capsule, hippocampus, thalamus and parietal cortex. Finally, intensities were inverted and normalized to an averaged intensity of all sections in a brain.

### Behavioral studies

Testing took place in a dedicated quiet room. Mice were allowed at least three days to acclimate after shipment. Test chambers were cleaned with 70% ethanol and aired for 3 min after each animal. Animal movements were registered and processed with ANY-maze software (Stoelting Co., Wood Dale, IL).

#### Open field test

Animals were placed individually in the center of an open clear field box (61 × 61 cm), and their spontaneous motor activity was recorded. The computer program automatically recorded the following parameters: mobile time, mean speed, traveled distance and time spent traveled in the center (40 x 40 cm) and peripheral zones.

#### Elevated plus maze test

The elevated plus maze, made of white acrylic plastic, consisted of four arms (each 28 × 5 cm) and a central area (5 × 5 cm) elevated 50 cm above the floor. Two arms were open and two were closed with 15-cm-high walls made of the same material. Mice were individually placed in the center facing an open arm and allowed to explore for 5 min. The following behaviors were scored: time spent in the closed and open arms.

#### Morris water maze test

Mice were placed in a circular 120 cm diameter tank with room temperature (22°C) water, which had been made opaque with the addition of non-toxic, white tempera paint. High contrast black and white images were placed on the walls of the testing room around the tank to serve as visual cues.

At the first stage of the test, the mice were trained to locate a visible 10 cm diameter platform exposed 1 cm above the water. If they did not find the platform within the 60-second trial limit, they were guided to it and allowed to stay for 20 seconds. The mice were wiped with towels after removal from the pool and placed under a heating lamp to dry. Five trials were performed and the platform location was changed for each trial.

At the second stage, mice were tested to find the hidden platform that was submerged 1 cm below the surface in the southeast quadrant. Mice were tested once a day for four consecutive days in the afternoon, five trials at a training session, at least 10 mins between the trials. Starting quadrants varied between the trials in the same order for all mice. The latency required to locate the platform and their path lengths was recorded.

The probe trial was performed on the 5^th^ day, where mice swam for 60 seconds with no platform in the tank. Time spent in the quadrant where the submerged platform had been in previous stages was recorded.

#### Contextual and cued fear conditioning test

The contextual and cued fear conditioning tests the ability of mice to learn and remember an association between environmental cues and aversive experiences. In this test, mice were placed into a conditioning chamber and were given parings of a conditioned stimulus (CS) (an auditory cue) and an aversive unconditioned stimulus (US) (an electric foot shock). The conditioning chamber consisted of opaque acrylic plastic 30x30x21 cm with a clear lid and a shocking grid on the floor made of 2 mm diameter metal rods, 6 mm between runs. During the conditioning stage on day 1, mice were allowed to freely explore the chamber for 120 seconds. Thereafter, a white 55 dB noise auditory cue was presented as a CS for 30 seconds, and a 0.8 mA foot shock was given to the mice as an US continuously during the last 2 seconds of the sound. The presentation of CS-US was repeated three times per session (120, 240, and 360 seconds after the beginning of the conditioning). Following the final foot shock, the mice were left undisturbed in the chambers for 90 seconds.

After the conditioning session had been completed, the mice were returned to the same conditioning chamber 24 h later and scored for freezing behavior to measure contextually conditioned fear (context test). The mice were placed in the conditioning chamber and were allowed to freely explore the chamber for 300 seconds without CS and US presentations. Cued test was conducted on the same day two hours after the context test. In this test, the shocking grid was removed and the walls of the chamber were covered with checkerboard pattern wallpaper, providing a novel context that was unrelated to the conditioning chamber. Mice were placed into the testing chamber for 3 min. At the end of the first 3 min, the CS auditory cue that had been presented at the time of conditioning was given to mice for 3 min. The fear conditioning chamber was wiped with 70% alcohol after each test. Fear memory was assessed based on freezing behavior to the conditioned cued or the contexts to which mice were previously exposed. The outcome variables were freezing time in the context test and during the first and last 30 seconds of the cued test.

#### Social interaction test

Two social behaviors (social interaction and social memory/novelty recognition) were quantified using a rectangular 3-chamber test that included a 20x45x30 cm middle chamber made of acrylic plastic, with 2 10x10 cm openings leading to two separate (left and right) chambers of the same size, each containing a steel cage enclosure. Each mouse (experimental subject) was placed in the middle chamber and allowed to explore and interact for 10 minutes, with the right chamber empty but an unfamiliar congener (Stranger I) (non-littermate control SPF mouse of the same gender, housed in a separate container) held in the steel cage enclosure in the left chamber. Social interaction was determined by measuring the time spent by the experimental subject in the chamber holding the unfamiliar congener versus the right chamber containing empty enclosure. To measure social memory (or novelty recognition), a new novel stimulus mouse (Stranger II) was subsequently placed in the previously empty right chamber. The tested mouse was allowed to explore and interact for 10 min. The stages of the test followed each other without delay. The same parameters as above were measured to determine the preference of the experimental subject for Stranger I or Stranger II. The social chamber was wiped with 70% alcohol after each test.

### Stereological estimation of neuron and oligodendrocyte populations

After MRI, brains were extracted from skulls, cryoprotected in 30% sucrose and frozen on dry ice. Serial 20 micron thick sections were cut 400 microns apart on a cryostat and mounted onto poly-L-lysine-coated slides (Sigma-Aldrich, St Louis, MO). Adjacent series were used to stain for neuronal and oligodendrocyte markers and myelin contents.

The sections used for immunostaining were blocked with 3% goat serum followed by incubation with the primary antibodies overnight at 4°C. This was followed by incubation with biotinylated secondary antibodies for 1 h at 21°C and avidin–biotin complex (Vectastain Elite ABC Kit, Vector Laboratories, Burlingame, CA) for 1 h. Color was developed using 3, 30-diaminobenzidine (Sigma-Aldrich). Primary antibodies used were rabbit anti-NeuN (ab177487, Abcam, 1:400) and mouse anti-Olig2 (MABN50, Millipore, 1:400). Secondary antibodies were goat anti-rabbit BA-1000 (1:200) and BA-9200 goat anti-mouse (1:200), both from Vector Laboratories. Labeled sections were visualized under a microscope (Leica Microsystems, Wetzler) attached to a motorized stage. An optical fractionator probe in StereoInvestigator software (MBF Bioscience, Williston, VT) was used to obtain an unbiased estimate of the total number of cells or cell density. Since exact boundaries of motor cortex are difficult to outline precisely, in order to estimate total neuron number in the region neuronal density in the motor cortex was estimated in 4 consecutive sections labeled with anti-NeuN from each brain sample starting 2 mm from the front of cerebrum. Boundaries of the motor cortex were outlined with a 5x objective and the cell counting was performed with a 40x objective. The counting frame size was 100 μm x 100 μm and the grid size was 600 μm x 600μm. Following the completion of counting for each section from a brain sample, cell density was computed using the estimated population and the volume of each counted area, provided by the software, and averaged for four sections.

To estimate the total number of neurons in the hippocampus, NeuN–positive cell counting was performed on all serial sections where the hippocampus was present, typically 4–5 per brain. The boundaries of the hippocampus sub-division were carefully defined using a mouse brain atlas [39] and by the clear morphological indication of conspicuous smaller and more densely organized CA1 pyramidal neurons, as compared to the relatively larger and less packed neurons characterizing the CA2-CA3 subdivisions [40]. Boundaries of the CA1, CA2/3 and dentate gyrus regions cortex were outlined with a 5x objective and the cell counting was performed with a 40x objective. The counting frame size was 50 μm x 50 μm and the grid size was 250 μm x 250μm. Estimated cell population number was computed using the stereological formula.

Oligodendrocyte density was estimated on a single slice in StereoInvestigator by placing a counting grid with the same parameters as above for NeuN at 0 bregma for striatum and cortex regions, -0.94 bregma for fimbria, and -1.28 bregma for corpus callosum and internal capsule regions. Oligodendrocyte density was obtained by dividing estimated population number by measured volume, provided by StereoInvestigator.

### Statistical analysis

Data are presented as means ± standard error of means. Comparison across treatment and gender groups for each age was made with two-way ANOVA. Post-*hoc* group comparison was performed by Tukey-Kramer method. A False Discovery Rate (FDR)-adjusted *p*-value (or q value) was also calculated using the Benjamini–Hochberg procedure to correct for multiple comparisons in MRI volumetric and MPF data. Latency time to platform in Morris water maze test was analyzed with repeated measures (RM) ANOVA with training day as the repeated factor and treatment group as the fixed factor. For comparison of outcome measures in behavioral tests, a predefined limited number of outcome measures (<4) was used and no correction for multiple comparisons was made [41]. The differences were considered significant at α = 0.05.

## Results

### Magnetic resonance imaging

#### Regional volume changes

**Fig 2:**
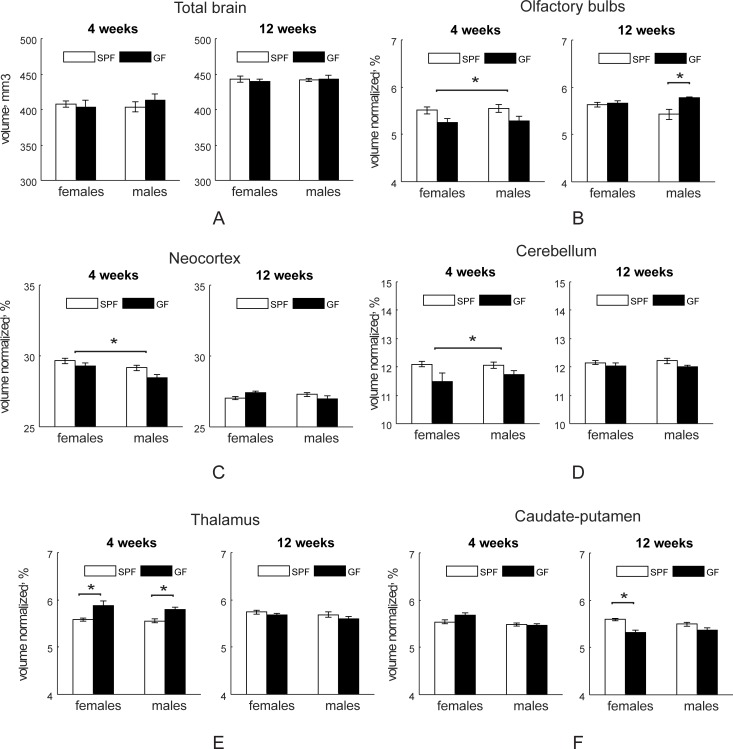
Regional volume differences between SPF and GF Mice. Regional volume differences in SPF and GF mice at four (n = 15 for both female and male SPF mice; n = 6 for female and n = 9 for male GF mice) and 12 (n = 13 for both female and male SPF and n = 7 for both female and male GF mice) weeks of age. Asterisks indicate significant differences when *p*-value is at least <0.05 between the treatment groups or between the individual groups in post-*hoc* comparisons where treatment group factor of two-way ANOVA for each testing age was significant.

Regional brain volumes obtained from *in vivo* atlas based brain parcellation methodology based on magnetization transfer tissue contract are summarized in absolute volume units in S1 Table and in percentage, normalized to individual mouse brain, in S2 Table. There was no difference in total brain volume between SPF and GF mice at either four or 12-week testing (Fig 2A). Relative gray matter volume was higher in SPF mice in olfactory bulbs, neocortex and cerebellum (Fig 2B–2D), but was lower in thalamus (Fig 2E) and forebrain/septum (S2 Table) relative to GF mice at four weeks. Olfactory bulbs size was different at 12 weeks in absolute volume (S1 Table) and post-*hoc* test revealed that relative olfactory bulb size was significantly enlarged in male GF mice (Fig 2B). At 12 weeks of age female SPF mice had significantly higher striatum volume (F_1, 30_ = 21.19, *p*<0.001) than GF mice on post-*hoc* comparisons (*p* = 0.0003, Fig 2F).

#### Local brain volume changes on tensor based morphometry

**Fig 3:**
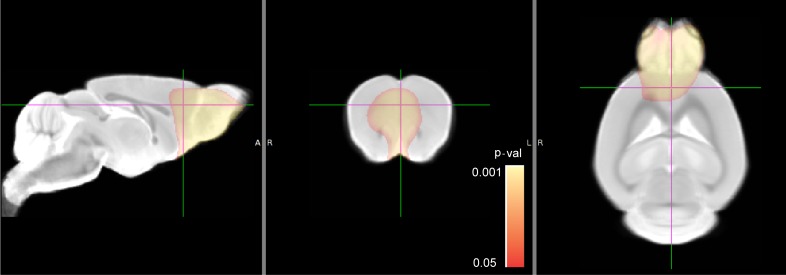
Tensor based morphometry comparison between SPF and GF mice. Animal numbers: SPF (n = 5 for both females and males) and GF (n = 5 for both females and males) mice at 12 weeks of age. No significant difference was detected at four weeks of age with the same number of animals in each group. The pseudo-colored statistical parametric map is overlaid on a gray scale template. Statistically significant regional volume expansion in olfactory bulbs and prefrontal cortex in GF group is indicated by pseudo-colored voxels in red-yellow scale. Color bar indicates corrected *p*-values range and *p* value of <0.05 was considered statistically significant.

Tensor-based morphometry (TBM) is a statistical mapping method based on a color-coded Jacobian determinant value developed to quantify regional structural differences relative to the corresponding anatomical template [42, 43]. No significant difference was found between the groups at four weeks. Significant regional expansion of the brain in GF mice was found in olfactory bulbs and prefrontal cortex at 12 weeks (Fig 3).

#### White matter organization and myelination indicated by fractional anisotropy

**Fig 4:**
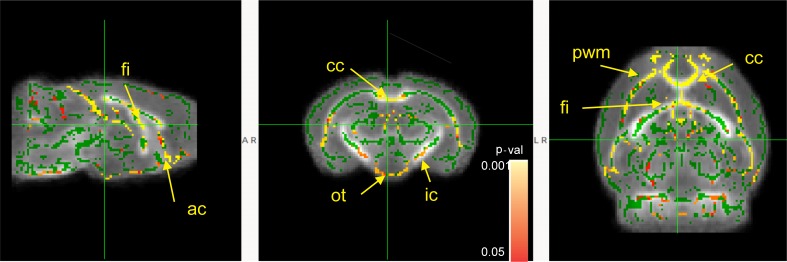
Statistical parametric maps of comparisons between SPF and GF mice using FA and radial diffusivity metrics. Animal numbers: SPF (n = 5 for both females and males) and GF (n = 5 for both females and males) mice at four weeks. No significant difference was detected at 12 weeks of age with the same number of animals in each group. Green skeleton lines were overlaid on gray scale FA, shown on sagittal, coronal and horizontal sections. Statistically significant voxels, where the parameters were less in the GF group, are depicted in red-yellow scale for FA. Color bar indicates corrected-*p*-values range and *p* value of <0.05 was considered statistically significant. Abbreviations of anatomic structures: ac-anterior commissure, cc-corpus callosum, ic-internal capsule, fi-fimbria, ot-optic tract, pwm-periventricular white matter.

SPF mice had significantly increased fractional anisotropy in fimbria, anterior commissure, corpus callosum, optic tract, internal capsule, and periventricular white matter at four weeks testing (Fig 4). No significant differences between SPF and GF mice were found at 12 weeks.

#### Macromolecular proton fraction (a biomarker of myelination) imaging

**Fig 5:**
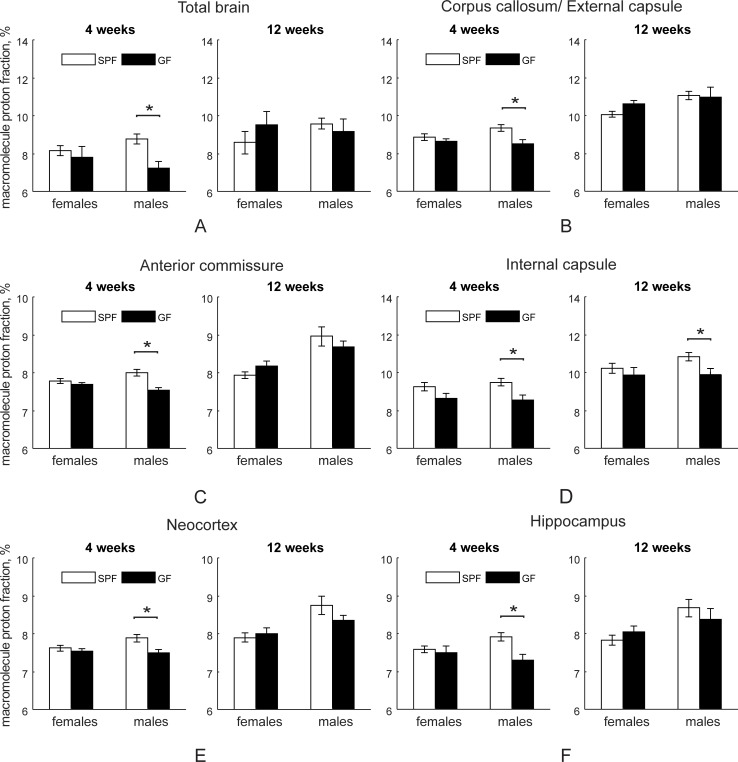
Macromolecule proton fraction (in percentage) in SPF and GF mice. Animal numbers at four (n = 15 for both female and male SPF mice; n = 6 for female and n = 9 for male GF mice) and 12 (n = 13 for both female and male SPF and n = 7 for both female and male GF mice) weeks of age. Asterisks indicate significant differences when *p*-value is at least <0.05 in between the groups in *post-hoc* comparisons where treatment group factor of two-way ANOVA for each testing age was significant.

MPF group values are summarized in S3 Table. Male SPF mice had increased MPF at four weeks in total brain (F_1,38_ = 6.51, *p* = 0.015), in white matter, including corpus callosum, anterior commissure and internal capsule (Fig 5A–5D), as well in gray matter structures including neocortex (Fig 5E), hippocampus (Fig 5F), hypothalamus and brainstem/midbrain (S3 Table). Gender factor and gender x group interaction factors were not significant at four weeks. At 12 weeks testing, MPF in SPF males was higher in internal capsule (Fig 5D) and gender factor was significant for all parceled regions (S3 Table).

### Myelin contents by Luxol fast blue staining

**Fig 6:**
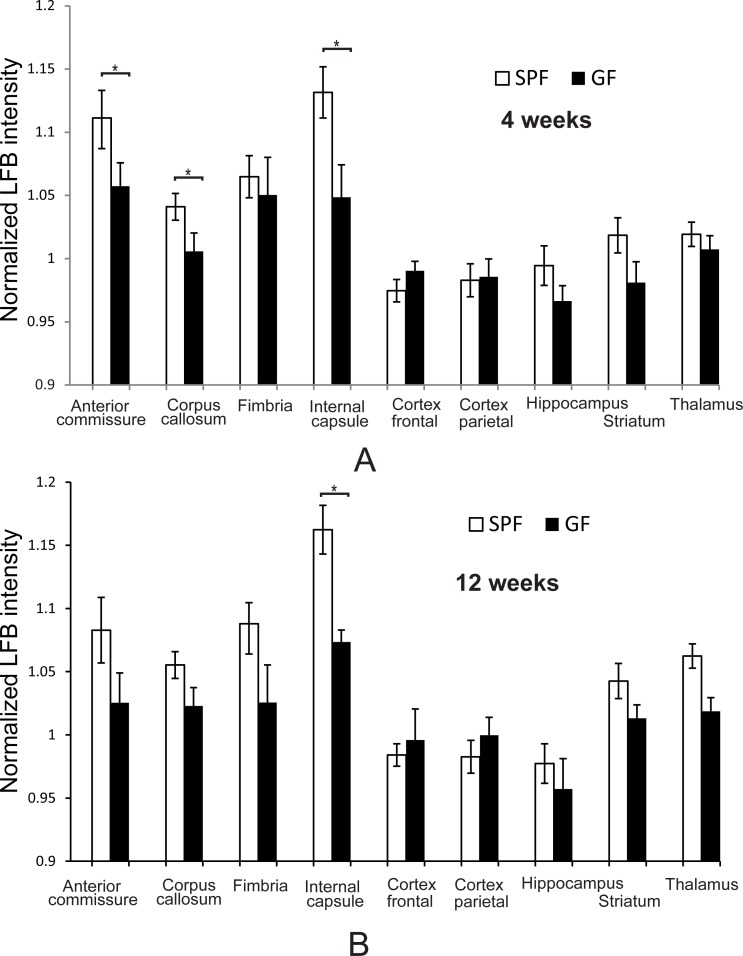
Myelin content determination by Luxol fast blue stain. Animal numbers at four (n = 6 for both SPF and GF mice) (A) and 12 (n = 6 for both SPF and GF mice) weeks of age (B). Asterisks indicate significant differences when *p*-value is at least <0.05 between SPF and GF group.

Intensity of LFB staining was significantly higher in SPF mice when compared to GF mice (both genders combined) in anterior commissure, corpus callosum, and internal capsule at four weeks of age (Fig 6A) and in internal capsule at 12 weeks of age (Fig 6B).

### Behavioral tests

#### Open field test and elevated plus maze tests

**Fig 7:**
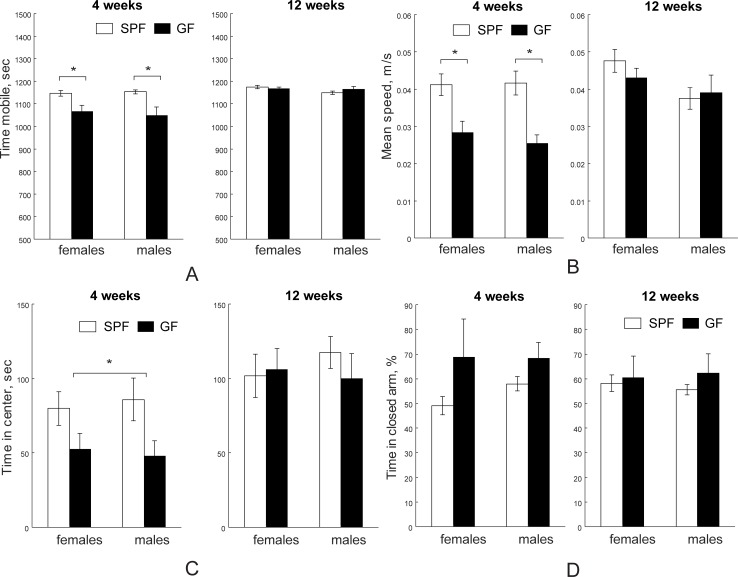
Open field and elevated plus maze behavioral tests. SPF mice were more mobile (A) and traveled with faster speed (B) at four weeks (n = 15 for both female and male SPF mice; n = 6 for female and n = 9 for male GF mice), but not at 12 weeks (n = 13 for both female and male SPF and n = 7 for both female and male GF mice). SPF mice spent more time in the center quadrant in the open field test at four weeks, but not at 12 weeks (C). D. GF mice were not different from SPF mice at four weeks and at 12 weeks in time spent in closed arm in elevated plus maze. Asterisks indicate significant differences when *p*-value is at least <0.05.

The effects of microbiota on neuronal general locomotor activities and anxiety-like behaviors were evaluated using open field test (OFT) and elevated plus maze (EPM). The key brain macrostructure that contribute to the neural circuits of anxiety are, but not limited to, amygdala, the bed nucleus of the stria terminalis, the ventral hippocampus and the prefrontal cortex. SPF mice were significantly more mobile (Fig 7A, F_1,39_ = 16.85, *p*< 0.001, two-way ANOVA) and had higher mean speed (Fig 7B, F_1,39_ = 14.95, *p*< 0.001, two-way ANOVA) than GF mice at four weeks in the OFT. No difference was found in gender factor and microbiota x gender interaction at four weeks. On post-*hoc* comparison, four-week old SPF females and males had significantly higher mobile time (*p* = 0.002 and *p* = 0.043, respectively) and mean speed (*p* = 0.0049 and *p* = 0.016, respectively) than GF females and males. As a result, SPF mice traveled significantly more distance (48.40±2.49 m) than GF mice (30.72±2.06 m) at four weeks (F_1, 39_ = 15.37, *p*< 0.001, two-way ANOVA). No difference was found in gender factor and microbiota x gender interaction in travel distance at four weeks of age. On post-*hoc* comparison, four weeks old SPF females and males were significantly different than GF females and males (*p* = 0.0048 and *p* = 0.018, respectively). Furthermore, four weeks old SPF mice spent significantly more time in center area (Fig 7C, F_1, 39_ = 4.26, *p* = 0.045, two-way ANOVA). No difference in mobile time, mean speed, traveled distance or time spent in the center area was found at 12 weeks testing. In the EPM test, no difference in time spent in closed arms was found in gender, microbiota, or microbiota x gender interaction at either four weeks or 12 weeks of age (Fig 7D).

#### Morris water maze

**Fig 8:**
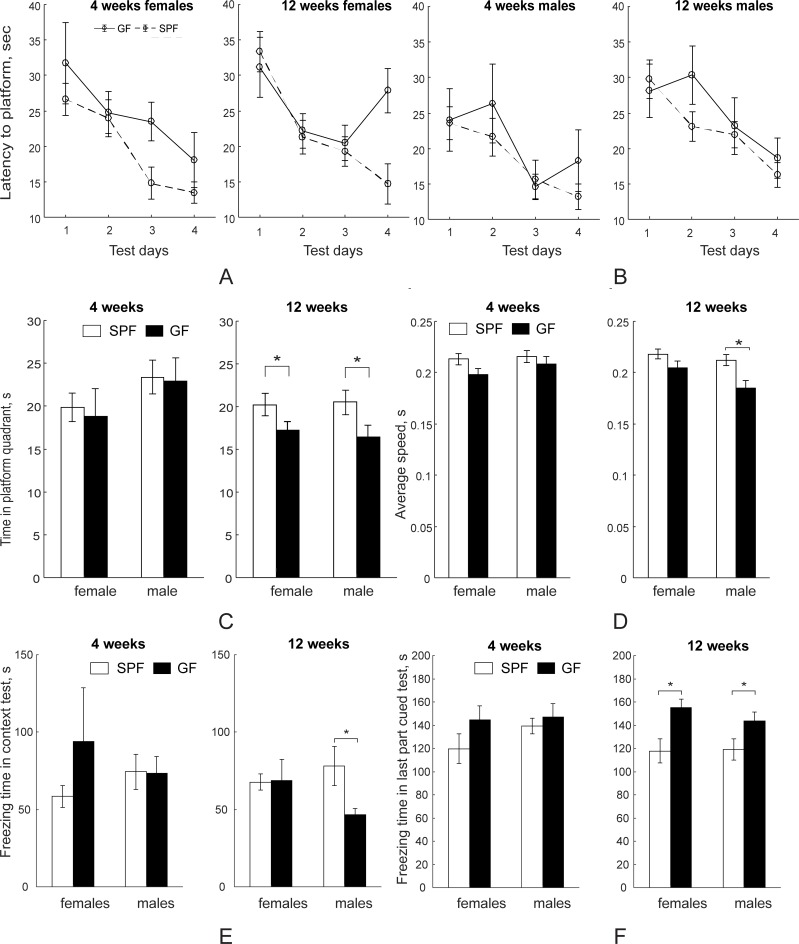
Memory and learning behavioral tests using Morris water maze and fear conditioning. No significant difference during training was found between SPF (n = 15 for both females and males) and GF (n = 6 for females and n = 9 for males) mice at 4 weeks (A), but at 12 weeks (B) SPF (n = 13 for both females and males) mice had significantly higher learning curve slopes than GF (n = 7 for both females and males) mice by RM ANOVA. (C). SPF mice spent significantly more time in the platform quadrant during the probe trial of the Morris water maze test at 12 weeks, but were not different from SPF mice at 4 weeks. (D). SPF males swim speed was significantly higher than GF males at 12 weeks. (E). Freezing time was longer in contextual fear conditioning test in male SPF mice at 12 weeks. (F). Freezing time was shorter for SPF mice in the second part of cued fear conditioning test (different chamber and sound cue presented) at 12 weeks. Asterisks indicate significant differences when *p*-value is at least <0.05.

Spatial learning and memory which are strongly associated with hippocampal circuitry as well as prefrontal cortex, the cingulate cortex, and the neostriatum [44] were evaluated using the Morris water maze in both SPF and GF mice. In testing latency to find the platform during training days by two-way ANOVA with one repeated measures (stage), training day factor was significant at both four weeks (F_3, 102_ = 11.72, *p*<0.001) and 12 weeks (F_2, 102_ = 4.8, *p*<0.001), demonstrating that in both SPF and GF groups the latency time to find the hidden platform decreased during training and both groups were learning (Fig 8A and 8B). The microbiota factor was not significantly different at four weeks, but was significant at 12 weeks (F_1, 36_ = 11.72, *p* = 0.035) during training between SPF and GF mice. Post-*hoc* analysis revealed that female SPF had shorter latencies to find the platform at the end of the training (day 4) (F_1, 16_ = 12.09, *p* = 0.003).

The probe trial where the platform had been removed after the training stages revealed no significant difference between the SPF and GF mice in time spent in the quadrant at the four-week time point, neither for males nor for females (Fig 8C). At 12 weeks testing SPF mice spent significantly more time in the quadrant than GF mice (Fig 8C, F_1, 36_ = 11.76, *p* = 0.0015, Two-way ANOVA) and in both genders by post-*hoc* comparisons.

No difference in average speed was found at four weeks testing (Fig 8D). At 12 weeks of age SPF mice had significantly higher average swimming speed than that of GF mice (F_1, 36_ = 11.72, *p* = 0.0016, two-way ANOVA). On post-*hoc* comparison, 12-week old SPF males had higher average speed that GF males (Fig 8D, *p* = 0.002).

#### Fear conditioning test

To characterize the hippocampal-dependent and/or amygdala-dependent long-term memory in SPF and GF mice, mice were subjected to contextual/cued fear conditioning. No difference in freezing time between SPF and GF mice in either gender was found before conditioning. There was no difference in freezing time between SPF and GF mice in contextual fear conditioning (changed environment and no acoustic CS presentation) at four-week testing (Fig 8E). However, SPF had significantly longer freezing time than GF mice at 12 weeks (F_1, 39_ = 11.44, *p* = 0.022) with specifically significantly more freezing time in male SPF mice (post-*hoc* test, *p* = 0.032). Gender factor was significant (F_1, 39_ = 11.44, *p* = 0.002) due to significantly lower freezing time in SPF males (*p* = 0.047) at four weeks of age. At 12 weeks of age, the freezing time of SPF mice in this stage was significantly longer than that of GF mice (F_1, 39_ = 11.44, *p* = 0.002). Post-*hoc* test revealed that the freezing time of SPF males was significantly longer than that of GF males (*p* = 0.047). No difference in freezing time in the second part of the cued fear conditioning test (changed environment and acoustic CS presentation) was found at 4 weeks of age, but at 12 weeks of age the freezing time of SPF mice was significantly shorter than in GF mice (F_1,39_ = 9.28, *p* = 0.0043, Fig 8F). Post-*hoc* test revealed that the freezing time of SPF was significantly shorter than GF mice (*p* = 0.047) in both genders.

#### Social interaction tests: Sociability and novelty

**Fig 9:**
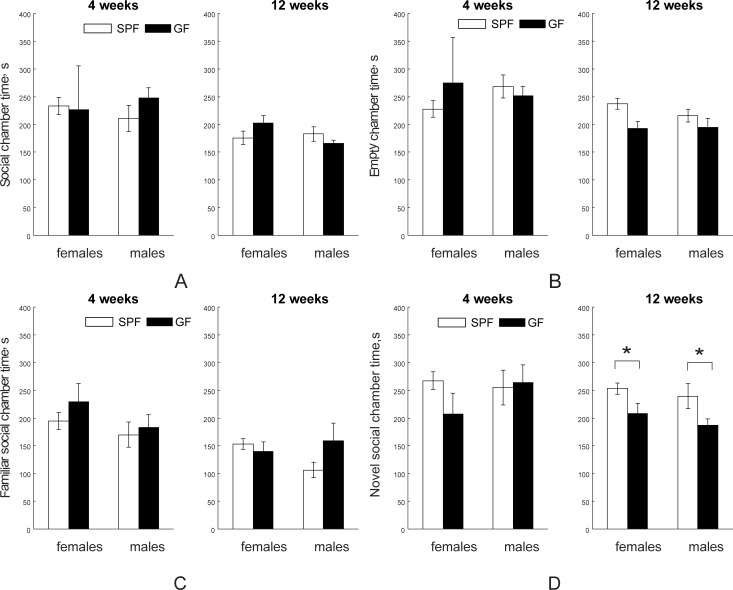
Social interaction tests using three-room chamber. In a sociability test SPF and GF mice were not different in time spent in chamber with a strange mouse (A) or with an empty cage (B) at either four (n = 15 for both female and male SPF mice; n = 6 for female and n = 9 for male GF mice) or 12 (n = 13 for both female and male SPF and n = 7 for both female and male GF mice) weeks of age. In the test for social novelty, no difference between SPF and GF groups was found in time spent with a familiar mouse (C), but there was a significant decrease in time spent with a novel mouse in GF mice at 12 weeks (D). Asterisks indicate significant differences when *p*-value is at least <0.05.

We further investigated whether there is a difference between SPF and GF mice in social interactions in the three-chamber social test, reflecting mostly prefrontal cortex and myelination plasticity. In the sociability test for social versus empty cage preference, no significant differences between SPF and GF mice in time spent in chamber with mice (Stranger 1, Fig 9A) or with empty cage were found either at four- or 12-week testing age (Fig 9B, with discrimination index shown in Figure A in S1 Fig). In the test for social novelty, no difference between SPF and GF group was found in time spent with a familiar mouse (Fig 9C), but SPF mice spent significantly more time with a novel mouse (Stranger 2) than GF mice in both genders at 12 weeks (F_1,32_ = 5.10, *p* = 0.030), (Fig 9D, with discrimination index shown in Figure B in S1 Fig).

### Stereological estimation of neurons and oligodendrocyte number

**Fig 10:**
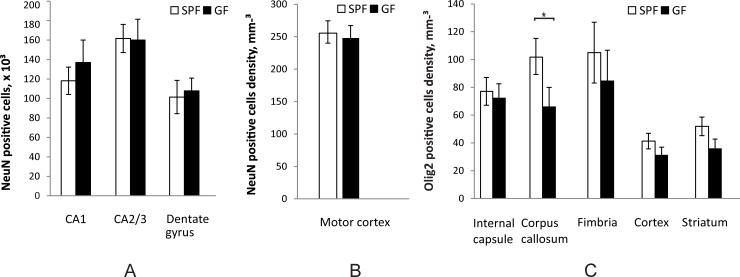
Stereological estimation of cell numbers between SPF and GF mice. (A). Estimation of neuron number in hippocampal regions at 12 weeks (n = 6 for both SPF and GF mice) of age. (B). Neuronal density in motor cortex at four weeks. (C). Oligodendrocyte density in white and gray matter regions at 12 weeks of age. Asterisks indicate significant differences when *p*-value is at least <0.05.

In an attempt to explain the difference in locomotion at four weeks and in spatial memory at 12 weeks, we estimated neuronal numbers in motor cortex and hippocampus and oligodendrocyte numbers in cortex and several regions of the white matter. Number of neurons identified by neuronal nuclear antigen (NeuN) was estimated for the entire hippocampus. No difference was found between SPF and GF mice in either CA1, CA2/3 or dentate gyrus of the hippocampus at 12 weeks (Fig 10A). Since exact boundaries of motor cortex are ambiguous to define, neuronal density was estimated in the central portion of the motor cortex. No significant difference in neuronal density was found between SPF and GF mice at four weeks of age (Fig 10B). Density of oligodendrocytes, determined by the Olig2 marker of oligodendrocytes lineage, was significantly higher in the corpus callosum of SPF mice at 12 weeks (*p* = 0.018) (Fig 10C), with a trend of increase in internal capsule, fimbria, cortex and striatum that did not reach significant difference.

## Discussion

**Fig 11:**
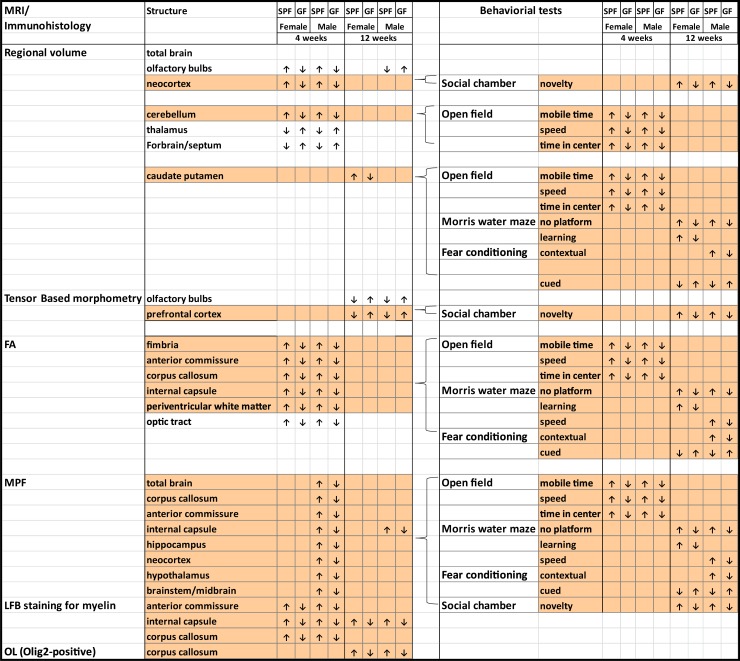
Summary of differences in structures and behaviors between SPF and GF Mice. Figure links the morphologic changes of regions in the brain with correlated behaviors.

The current study contributes to the rapidly growing body of literature related to the role of the gut microbiota and gut-brain interactions in shaping brain development and behaviors. Novel aspects of the study were to apply a combination of function-focusing modalities (advanced MRI neuroimaging and behavioral tests) with complementary immunohistological studies to examine the effects of commensal bacteria on brain structure and behaviors in C57BL/6J mice. Furthermore, novel quantitative macromolecule fraction MRI mapping was used to evaluate the impact of commensal bacteria on myelination across multiple brain structures. Both *in vivo* and *ex vivo* MRI revealed that SPF mice with commensal bacteria demonstrated several larger regions in grey matter and other brain regions than GF mice without commensal bacteria. We furthered observed SPF mice were more myelinated in grey matter structures including neocortex, hippocampus and several major white matter tracts in juvenile male mice at four weeks of age and in the internal capsule in males at 12 weeks of age. These differences in myelination were verified by Luxol fast blue staining for myelin. More importantly, the observed brain structure differences correlated to differences in behaviors between SPF and GF mice (Fig 11). We demonstrated that SPF and GF mice display transient differences in anxiety-related behaviors, and long-term differences in spatial memory, contextual and cued memory, and social novelty. The volumetric and organization differences between SPF and GF mice observed in fimbria, corpus callosum, internal capsule and dorsal striatum development might explain the difference in motor and cognitive outcomes in open field, Morris water maze, and fear conditioning tests; whereas the volumetric and myelination differences in neocortex and prefrontal cortex might contribute to the difference in social behaviors. Therefore, by linking affected specific regions of the brain to associated functions, our study provides evidence that commensal bacteria can influence brain development and behaviors.

First, our study demonstrated that microbiota influence the development of volumes of diverse brain structures. MRI is widely used in neurological development studies as it can non-invasively quantify brain structural changes and correlate with motor and cognitive development in clinical trials[11] and animal studies[13, 45]. Studies of normal brain maturation in humans report volumetric changes of grey matter and white matter between childhood and adolescence but relatively stable total brain volume during this period [14, 46]. Similarly, in mice, total brain volume by MRI has been found to reach stability at three weeks, but grey matter and white matter maturation including myelination continuously progresses during the first three months [47]. Our study, to our knowledge, for the first time, systematically investigated the role of microbiota in volumetric differences. Both automatic brain parcellation *in vivo* and tensor based morphometry *ex vivo* MRI were used. The differences were observed mostly in the early post-weaning period. SPF mice had transiently higher volume of cortex, cerebellum, olfactory bulbs and a decrease in thalamus and forbrain/septum volume compared to GF mice at four weeks of age. These observations agree with the notion [48] that early gut microbiota is important for early brain development.

Our study next investigated the impact of microbiota on white matter development. DTI is a frequently used neuroimaging method to evaluate white matter integrity and utilizes derived index of fractional anisotropy (FA) as a surrogate measure for white matter organization and myelination. Changes in FA values can assess the maturation of white matter reflecting alterations in axonal number, myelin structure integrity, and axonal cytoskeleton integrity [49]. In our study, we detected significantly higher FA values in the fimbria, corpus callosum, internal capsule, periventricular white matter and optic tracts, in both male and female SPF mice at four weeks compared to the GF mice. A large body of literature connects differences in FA with behavioral outcomes. In animal studies, fimbria-fornix (fringe of hippocampus) lesions altered spatial learning/memory and locomotion [50, 51]. In human studies, FA values of several white matter tracts including fimbria and corpus callosum have been shown to correlate positively with fine motor and cognitive scores of Bayley Scales of Infant and Toddler Development in children born prematurely and studied at two years of corrected age [52]. The corpus callosum coordinates sensory, motor, cognitive, and emotional functions from both hemispheres in infants [53]. FA values of corpus callosum have been shown to be positively associated with cognitive outcomes [54–56]. FA values of the internal capsule [57] are associated with better connectivity and motor skills in adolescents born prematurely[58] and cognitive levels in periventricular leukomalacia patients [54] [59, 60]. FA values of periventricular white matter are positively correlated with white matter development and cognitive functions in the normal pediatric population [61] and can predict white matter injury in several prematurity-related conditions [58]. Consistent with this literature, the microbiota dependent differences we find in FA values of fimbria, corpus callosum, internal capsule and periventricular white matter might explain the differences in motor activity in the open field test, spatial memory in the Morris water maze and contextual memory in the fear conditioning test in our study.

One of the major findings of this study is the previously undescribed microbiota dependent-hypomyelination of several gray matter structures including neocortex, hippocampus, brainstem and major white matter tracts including the corpus callosum, anterior commissure, and internal capsule specifically in GF mice using MPF imaging. Both cerebral and white matter myelination provide an assessment of the maturation of the brain [18, 62, 63] and the status of myelination of the brain is a strong indicator of postnatal neurologic functional maturity [64]. In our study the difference was mostly significant at four weeks but not at 12 weeks with the exception of internal capsule. LFB staining agreed with the MPF data in most of the white matter areas, particularly confirming the higher degree of myelination in SPF mice when compared to GF mice in the internal capsule at 12 weeks. The finding of cortical hypomyelination at four weeks in our study in GF mice is in contrast to previously reported hypermyelination of prefrontal cortex found at 10 weeks in Swiss Webster mice [21]. The prefrontal cortex integrates external stimuli and controls several domains of complex behavior [65]. We did not find differences between SPF and GF mice in myelination specifically in prefrontal cortex using MRI and LFB staining at 12 weeks. Discrepancies between our findings and others can be explained by the different mouse strains and techniques used to assess myelination. Use of MPF on MRI and LFB staining, both of which are imaging techniques, in our study ensures spatial specificity of gray and white matter sampling. Disturbances in myelination in young GF animals suggest the importance of commensal microbiota on brain myelination and extend the impact of early microbial colonization on brain maturation.

There have been several studies recognizing the differences in behaviors between SPF and GF mice, especially on risk-avoidance and exploratory tasks, learning and memory, and social behaviors [4]. However, current literature data are not consistent on behaviors in different strains of mice. To date, in BALB/c, NMRI and Swiss Webster mouse strains, decreased exploratory behaviors in SPF mice were reported using open field and elevated maze tests [6, 8, 20, 66] while C57BL/6N SPF mice displayed increased anxiety in the light/dark preference test but decreased anxiety in the step-down test compared to the GF mice [67]. Even within the three substrains of C57BL/6 mice, the C57BL/6J, C57/BL6N, and C57BL/6C, C57BL/6J mice displayed the most exploratory behaviors in the open field and elevated plus maze test among the three substrains [68]. In our studies, we reported in the open field test that SPF mice of the C57BL/6J strain expressed higher exploratory activities, and thus greater anxiolytic behaviors and locomotion at four weeks of age compared to GF mice. We speculate that the variations in these studies including ours can be attributed to differences in the genetic background and microbiome profiles in mouse strains. These findings reflect the complexity of microbiota and host bidirectional interactions while indicating that the microbiota indeed has a role in shaping host exploratory/anxiety behaviors.

Previously, decreased working memory tested by novel object recognition was found in female GF Swiss Webster mice [69] and antibiotic-treated SPF C57BL/6 mice [70]. Our data also reveal SPF mice have a better spatial memory at 12 weeks of age in the Morris Water maze test compared to GF mice. Early studies have shown that striatal lesions impair animal performance in spatial learning tasks [71–73]. Considering the role of the dorsomedial striatum in spatial-cognitive function, the decrease in striatum volume reported in this study might explain the spatial memory deficits in GF mice on the Morris water maze at 12 weeks of age. We also demonstrate that male SPF mice have better contextual memory in the cued fear conditioning test at 12 weeks of age which is hippocampus and frontal/ventromedial/cingulate cortex dependent [74]. However, they have reduced cued memory retention relative to GF mice, which is considered amygdala dependent [75]. A recent study from Hoban *et*. *al*. using C57BL/6N demonstrated that SPF mice had better cued memory retention, but were not different from GF mice in context recall [76]. The discrepancy between these findings might be explained by different mouse strains. Studies have shown that C57BL/6N mice have better contextual memory than C57BL/6J mice but no difference in cued memory between the two sub-strains was found [77]. The variation observed in studies could also be due to the experimental protocols applied. We used the traditional 24-hour retention protocol while Hoban *et*. *al*. used a modified six-hour retention protocol.

Social impairment in individuals with several neurological disorders such as attention deficit hyperactive disorder [78], autism [79], conduct disorder[80] and compulsive obsessive disorder [81] have been documented in children and into adulthood. Interestingly these disorders are usually associated with GI problems and dysbiosis in gut microbiota [82, 83]. We found no difference in sociability between C57BL/6J SPF and GF mice. However, SPF mice had a better preference for social novelty than GF mice with more profound effects found in males. Previous studies using Swiss Webster mice had conflicting results in sociability and social novelty in SPF mice compared to GF mice [10] [84]. The discrepancies between the previous studies were thought to be due, in part, to the differences in protocols, particularly use of different social partner strains and the age of the animals when tested.

Taken together, the different manifestations of behaviors in these studies including ours can be attributed to differences in the genetic background as well as differences in the microbiota of the different mouse strains [67, 85–87]. Other potential factors affecting the outcomes are the age of animals when tests are performed and the tests and protocols chosen [4]. Therefore, we argue that the interpretation of these studies should pay specific attentions to these factors. Future studies investigating the mechanisms by which the gut microbiota communicates with the brain are urgently needed.

There is a well-documented sexual dimorphism in behaviors in clinical and animal studies [88–91]. In humans, the prevalence of depression or anxiety is two to four times higher in women, whereas autism and ADHD are more common in men [88, 92]. In term of cognitive functions, women seem to do better on short term memory than men, whereas men perform better in spatial tasks [93–95]. In animals, C57BL/6J female mice have been previously reported to be more anxious in EPM test than the male counterparts but no difference was detected between the sexes in exploratory activities in the open field test [90]. There has also been no sociability and novelty difference between the female and male C57BL/6J mice in the three-chamber test [91]. In Morris Water Maze test, male rats show advantages in spatial learning, which is hippocampus-depended, and reference memory for rats independent of strains, protocols, ages and rearing environments, but mice show no sexual differences [96, 97]. Our data largely agrees with most of the reported studies in C57BL/6J mice where we did not find significant differences between genders (Figs 7–9). This prompts caution when translating animal model data to human behaviors when using specific strains of rodents. More carefully designed and controlled animal experiments are required to study sexual dimorphisms since many factors such as genetic wiring, hormonal stage, circadian rhythms, and different responses between female and male animals to training regime may influence the rodent behaviors. Our results, however, strengthen the notion that the differences in behaviors we observed in this study were driven by the presence or absence of microbiota. The area needed to be further explored is how the presence of different microbial communities or the absence of microbiota affects sexes differently. With most of the behaviors we observed not displaying sexual dimorphism, we specifically found that female GF mice had deficits in spatial learning and male GF mice had deficits in contextual memory. In one of the limited recent studies [98] colonizing early postnatal GF mice with human *Bifidobacterium* spp. improved recognition memory in both female and male mice but only restored the anxiety-like behaviors in female GF mice. The mechanisms of this sex-specific host behavior and microbiota interaction are needed to translate current findings to clinical applications.

Collectively, emerging preclinical studies have made microbiota a potential target to improve brain plasticity and functions. The current experimental approaches to investigate the effect of microbiota on brain functions include using prebiotics [99–101], probiotics [98, 102, 103], or antibiotics [23, 70, 104, 105] to manipulate gut microbiota as well as “humanized” germ free mouse models [9, 98, 106]. For example, human milk oligosaccharides 3′ Sialyllactose or 6′ Sialyllactose as a prebiotic treatment normalized stress-induced anxiety-like behaviors [101]. Feeding *Bifidobacteria breve* 1205 to innately anxious BALB/c mice reduced the general anxiety behaviors in EPM test [102]. Perturbation of gut microbiota by oral administration of antibiotics induced depression-like behaviors in tail suspension test and reduced social novelty in three-chamber social interaction test in C57BL/6J mice [104]. Although these studies explored changes of behaviors and molecular alteration in the brain due to microbiota manipulation, the knowledge of affected brain structure and organization was lacking. Our findings describe fundamental differences in brain structures and behaviors between SPF and GF mice to lay the groundwork for future studies to identify and potentially target brain structures and/or functions of interest for therapeutic development.

A limitation of the study was that although our animals were housed in positive-pressured isocages which have been shown to have the ability to maintain the identities of microbial communities in GF mice for up to six months in several studies [107, 108] therefore eliminating possible contamination during transport and housing, we did not maintain the microbiota free status of the GF mice during the nine-day behavioral testing time. We thus refer to these mice as “ex-GF.” It is unlikely that morphological changes would occur in the short testing period of nine days after GF mice first encountered the environment in our study. Other studies have shown that anxiety levels [67], social cognition impairment [10], and HPA stress response [9] were not affected during conventionalization of adult GF mice and in post weaning colonized GF mice, the time points used in this study. Furthermore, SPF mice in our study were subjected to the same “new” environment as the GF mice. Any effects due to this short encounter of a new microbial community should apply to both SPF and GF mice, not GF alone. Therefore, our results in brain development and behavioral differences between SPF and ex-GF mice can still be interpreted as an effect of commensal microbiota.

Taken together, the current study provides strong evidence in support of the notion that microbiota affects brain volume, white matter development, and myelination as well as anxiety, cognition and memory and social functions. The differences seen at four weeks, suggest an impact of the microbiota specifically on early brain development. There may be a developmental window when the effects of gut microbiota on brain development are the largest and then not structurally detectable at a later age. This emphasizes the importance of further imaging and behavioral studies on the effect of gut microbiota on early brain development.

## Supporting information

S1 Fig Social discrimination index. (PDF)

S1 Table Regional brain volumes, in mm^3^ in SPF and GF mice at four and 12 weeks of age. Data presented as mean ± standard error of mean. P-values represent results of two-way ANOVA for each testing age with treatment (SPF, GF) and gender (female, male) as factor and their interaction term. Bold italic font indicates if *p*-values<0.05. The value in parenthesis indicated *p*-values, corrected for multiple comparisons using False Discovery Rate (q = 0.05).
(PDF)

S2 Table Regional brain volumes normalized to the total brain volume for each mouse, in %, in SPF and GF mice at four and 12 weeks age. Data presented as mean ±standard error of mean. *p*-values represent results of two-way ANOVA for each testing age with treatment (SPF, GF) and gender (female, male) as factor and their interaction term. Bold italic font indicates if *p*-values<0.05. The value in parenthesis indicated *p*-values, corrected for multiple comparisons using False Discovery Rate (q = 0.05).
(PDF)

S3 Table Macromolecule proton fraction, in %, in SPF and GF mice at four and 12 weeks of age. Data presented as mean ±standard error of mean. P-values represent results of two-way ANOVA for each testing age with treatment (SPF, GF) and gender (female, male) as factor and their interaction term. Bold italic font indicates if *p*-values<0.05. The value in parenthesis indicated *p*-values, corrected for multiple comparisons using False Discovery Rate (q = 0.05).
(PDF)
