# Supplementary figures and images for: Microbiota influence the development of the brain and behaviors in C57BL/6J mice
Source: PLoS One. 2018 Aug 3;13(8):e0201829. doi: 10.1371/journal.pone.0201829 (PMC6075787; doi:10.1371/journal.pone.0201829)

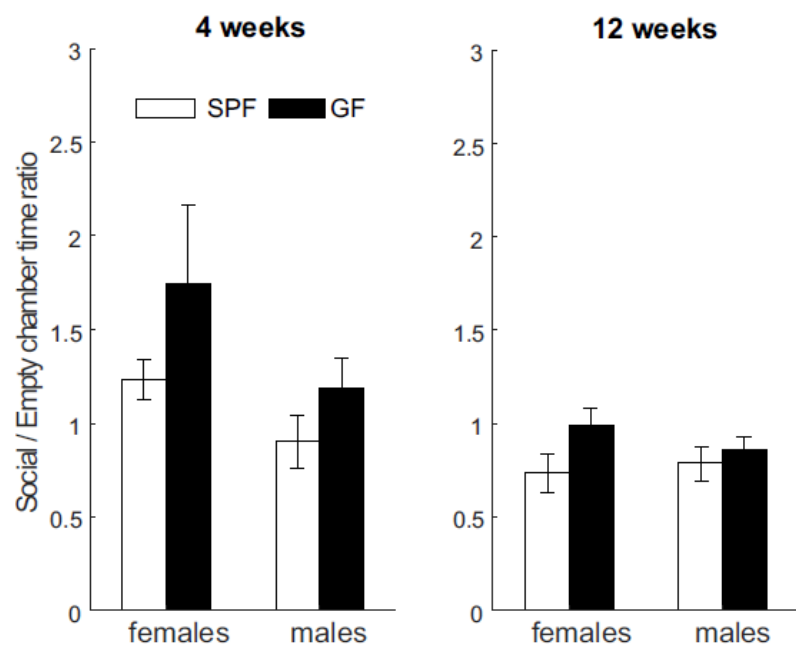

A

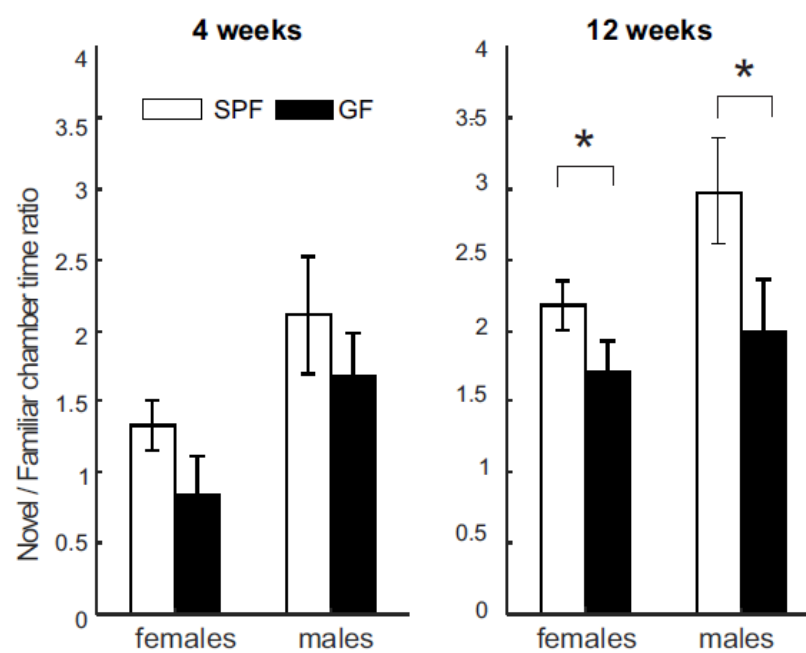

B

Supplement: S1 Fig — (PDF) [file pone.0201829.s001.pdf]
