# Supplementary material for: Microbiota influence the development of the brain and behaviors in C57BL/6J mice
Source: PLoS One. 2018 Aug 3;13(8):e0201829. doi: 10.1371/journal.pone.0201829 (PMC6075787; doi:10.1371/journal.pone.0201829)
Supplement: S1 Table — Data presented as mean ± standard error of mean. P-values represent results of two-way ANOVA for each testing age with treatment (SPF, GF) and gender (female, male) as factor and their interaction term. Bold italic font indicates if p-values<0.05. The value in parenthesis indicated p-values, corrected for multiple comparisons using False Discovery Rate (q = 0.05). (PDF) [file pone.0201829.s002.pdf]

Volume	4 weeks SPF		4weeks GF		*p*-value			12 weeks SPF		12 week s GF		*p*-value		
	female	male	female	male	treatment	gender	interaction	female	male	female	male	treatment	gender	interaction
Hippocampus	21.16± 0.33	20.54± 0.51	21.02± 0.55	21.43± 0.49	.495 (.72)	.848 (.969)	.345 (.646)	22.91± 0.35	23.32± 0.26	22.88± 0.51	22.71± 0.42	.435 (.733)	.767 (.877)	.483 (.844)
Corpus callosum/ external capsule	9.08± 0.20	8.62± 0.29	9.53± 0.08	9.48± 0.26	***.041 (.164)***	.403 (.812)	.508 (.646)	10.25± 0.34	9.98± 0.28	9.86± 0.31	9.80± 0.37	.458 (.733)	.668 (.877)	.791 (.844)
Caudate-putamen	22.62± 0.33	22.15± 0.43	22.91± 0.43	22.57± 0.43	.47 (.72)	.409 (.812)	.893 (.957)	24.77± 0.30	24.29± 0.23	23.38± 0.36	23.80± 0.43	***.01 (.16)***	.925 (.987)	.205 (.547)
Anterior commissure	5.94± 0.26	5.56± 0.30	6.95± 0.20	6.53± 0.30	***.007 (.112)***	.249 (.812)	.956 (.957)	6.70± 0.28	6.20± 0.34	6.43± 0.39	6.43± 0.37	.959 (.959)	.503 (.877)	.513 (.844)
Internal capsule	3.58± 0.16	3.22± 0.11	4.04± 0.68	4.03± 0.28	***.015 (.12)***	.457 (.812)	.487 (.646)	4.01± 0.14	3.64± 0.09	3.66± 0.12	3.89± 0.12	.743 (.959)	.638 (.877)	.048 (.384)
Thalamus	22.78± 0.32	22.45± 0.46	23.63± 0.24	23.94± 0.44	***.023 (.123)***	.989 (.997)	.525 (.646)	25.44± 0.23	25.15± 0.33	24.95± 0.26	24.85± 0.40	.242 (.553)	.551 (.877)	.774 (.844)
Cerebellum	49.38± 0.71	48.69± 0.99	46.39± 2.31	48.57± 1.45	.233 (.466)	.565 (.859)	.272 (.646)	53.85± 0.62	54.00± 0.52	52.93± 0.67	53.27± 0.40	.232 (.553)	.721 (.877)	.887 (.887)
Superior colliculi	7.44± 0.09	7.41± 0.14	7.46± 0.22	7.85± 0.17	.182 (.416)	.283 (.812)	.205 (.646)	7.92± 0.07	8.29± 0.16	8.25± 0.12	8.25± 0.13	.288 (.576)	.18 (.877)	.168 (.541)
Hypothalamus	11.82± 0.12	11.67± 0.19	11.56± 0.36	12.10± 0.28	.708 (.853)	.402 (.812)	.152 (.646)	12.78± 0.11	12.75± 0.24	12.82± 0.18	12.68± 0.16	.956 (.959)	.676 (.877)	.791 (.844)
Inferior colliculi	5.68± 0.09	5.30± 0.10	5.21± 0.14	5.59± 0.22	.573 (.764)	.997 (.997)	***.018 (.264)***	5.96± 0.09	6.09± 0.16	5.84± 0.13	5.85± 0.09	.201 (.553)	.596 (.877)	.658 (.844)
Neocortex	120.92± 1.23	117.67± 1.66	118.05± 2.72	117.57± 2.46	.483 (.72)	.38 (.812)	.513 (.646)	119.78± 1.22	120.65± 1.04	120.48± 0.72	119.63± 1.92	.906 (.959)	.996 (.996)	.538 (.844)
Amygdala	16.15± 0.21	15.50± 0.32	15.40± 0.57	16.39± 0.37	.851 (.853)	.644 (.859)	.033 (.264)	17.47± 0.38	17.79± 0.30	17.04± 0.47	17.69± 0.60	.555 (.807)	.283 (.877)	.715 (.844)
Olfactory bulbs	22.47± 0.36	22.38± 0.41	21.20± 0.82	21.85± 0.61	.11 (.352)	.619 (.859)	.507 (.646)	24.95± 0.30	23.99± 0.43	24.95± 0.22	25.65± 0.30	.043 (.292)	.738 (.877)	.042 (.384)
Brainstem	49.80± 1.41	53.34± 1.42	49.56± 3.08	52.90± 1.30	.853 (.853)	.067 (.812)	.957 (.957)	61.23± 0.95	61.00± 0.71	61.75± 1.63	64.69± 0.91	.073 (.292)	.24 (.877)	.169 (.541)
Fimbria	2.17± 0.06	2.25± 0.06	2.24± 0.25	2.43± 0.05	.167 (.416)	.119 (.812)	.524 (.646)	2.42± 0.03	2.55± 0.06	2.41± 0.06	2.35± 0.06	.071 (.292)	.548 (.877)	.117 (.541)
Total brain	408.04± 4.28	403.89± 6.78	402.91± 10.26	413.01± 8.79	.802 (.853)	.709 (.873)	.373 (.646)	442.91± 3.96	442.05± 2.12	439.74± 3.38	443.46± 4.72	.83 (.959)	.727 (.877)	.578 (.844)
