# Supplementary material for: Microbiota influence the development of the brain and behaviors in C57BL/6J mice
Source: PLoS One. 2018 Aug 3;13(8):e0201829. doi: 10.1371/journal.pone.0201829 (PMC6075787; doi:10.1371/journal.pone.0201829)
Supplement: S2 Table — Data presented as mean ±standard error of mean. p-values represent results of two-way ANOVA for each testing age with treatment (SPF, GF) and gender (female, male) as factor and their interaction term. Bold italic font indicates if p-values<0.05. The value in parenthesis indicated p-values, corrected for multiple comparisons using False Discovery Rate (q = 0.05). (PDF) [file pone.0201829.s003.pdf]

Volume Normalized	4 weeks SPF		4weeks GF		*p*-value			12 weeks SPF		12 week s GF		*p*-value		
	female	male	female	male	treatment	gender	interaction	female	male	female	male	treatment	gender	interaction
Hippocampus	5.19± 0.05	5.08± 0.06	5.23± 0.20	5.19± 0.06	.356 (.445)	.393 (.536)	.673 (.903)	5.17± 0.06	5.28± 0.06	5.20± 0.10	5.12± 0.05	.41 (.662)	.887 (.887)	.232 (.476)
Corpus callosum /external capsule	2.23± 0.05	2.13± 0.06	2.37± 0.05	2.29± 0.04	***.02 (.05)***	.182 (.396)	.869 (.94)	2.31± 0.06	2.26± 0.07	2.24± 0.06	2.21± 0.07	.441 (.662)	.584 (.782)	.923 (.923)
Caudate-putamen	5.54± 0.04	5.48± 0.04	5.69± 0.05	5.47± 0.03	.177 (.295)	***.006 (.065)***	.1 (.5)	5.59± 0.03	5.49± 0.04	5.32± 0.06	5.36± 0.05	***<0.001 (<0.001)***	.583 (.782)	.108 (.27)
Anterior commissure	1.46± 0.07	1.37± 0.07	1.73± 0.07	1.58± 0.07	***.007 (.035)***	.171 (.396)	.722 (.903)	1.51± 0.06	1.40± 0.08	1.46± 0.09	1.45± 0.08	.987 (.987)	.462 (.782)	.568 (.775)
Internal capsule	0.88± 0.04	0.80± 0.02	1.00± 0.16	0.98± 0.06	***.014 (.045)***	.372 (.536)	.631 (.903)	0.90± 0.03	0.82± 0.02	0.83± 0.03	0.88± 0.03	.776 (.97)	.587 (.782)	.062 (.27)
Thalamus	5.58± 0.03	5.56± 0.04	5.87± 0.11	5.80± 0.05	***.001 (.015)***	.38 (.536)	.652 (.903)	5.74± 0.04	5.69± 0.05	5.67± 0.03	5.60± 0.04	.144 (.36)	.219 (.782)	.892 (.923)
Cerebellum	12.10 ± 0.09	12.05± 0.11	11.49± 0.30	11.74± 0.12	***.003 (.023)***	.481 (.601)	.304 (.903)	12.16 ± 0.06	12.22± 0.11	12.04 ± 0.12	12.01 ± 0.05	.106 (.318)	.848 (.887)	.679 (.849)
Superior colliculi	1.82± 0.01	1.83± 0.01	1.85± 0.05	1.90± 0.02	***.04 (.086)***	.185 (.396)	.377 (.903)	1.79± 0.02	1.88± 0.03	1.88± 0.03	1.86± 0.02	.207 (.388)	.235 (.782)	.076 (.27)
Hypothalamus	2.90± 0.02	2.89± 0.03	2.87± 0.04	2.93± 0.03	.85 (.911)	.351 (.536)	.282 (.903)	2.89± 0.03	2.88± 0.05	2.92± 0.04	2.86± 0.04	.933 (.987)	.493 (.782)	.55 (.775)
Inferior colliculi	1.39± 0.02	1.31± 0.01	1.29± 0.03	1.35± 0.03	.231 (.315)	.636 (.734)	***.007 (.105)***	1.34± 0.01	1.38± 0.04	1.33± 0.02	1.32± 0.01	.182 (.388)	.651 (.782)	.471 (.775)
Neocortex	29.65 ± 0.21	29.16± 0.18	29.31± 0.22	28.48± 0.23	***.05 (.094)***	.013 (.065)	.503 (.903)	27.04 ± 0.13	27.29± 0.13	27.40 ± 0.11	26.97 ± 0.20	.902 (.987)	.551 (.782)	***.036 (.27)***
Amygdala	3.96± 0.05	3.84± 0.06	3.82± 0.08	3.97± 0.04	.936 (.936)	.82 (.82)	.053 (.398)	3.94± 0.06	4.03± 0.07	3.87± 0.10	3.99± 0.13	.565 (.77)	.27 (.782)	.865 (.923)
Olfactory bulbs	5.51± 0.07	5.55± 0.08	5.26± 0.07	5.29± 0.09	***.015 (.045)***	.728 (.78)	.981 (.981)	5.63± 0.05	5.43± 0.10	5.67± 0.05	5.78± 0.02	***.022 (.165)***	.558 (.782)	.063 (.27)
Brainstem	12.19 ± 0.28	13.19± 0.19	12.27± 0.46	12.81± 0.10	.597 (.689)	***.011 (.065)***	.432 (.903)	13.83 ± 0.20	13.80± 0.16	14.04 ± 0.38	14.59 ± 0.21	.051 (.255)	.304 (.782)	.254 (.476)
Fimbria	0.53± 0.01	0.56± 0.01	0.56± 0.06	0.59± 0.01	.202 (.303)	.156 (.396)	.877 (.94)	0.55± 0.01	0.58± 0.01	0.55± 0.01	0.53± 0.01	.093 (.318)	.678 (.782)	.099 (.27)
Total brain	100	100	100	100				100	100	100	100			
