# Supplementary material for: Microbiota influence the development of the brain and behaviors in C57BL/6J mice
Source: PLoS One. 2018 Aug 3;13(8):e0201829. doi: 10.1371/journal.pone.0201829 (PMC6075787; doi:10.1371/journal.pone.0201829)
Supplement: S3 Table — Data presented as mean ±standard error of mean. P-values represent results of two-way ANOVA for each testing age with treatment (SPF, GF) and gender (female, male) as factor and their interaction term. Bold italic font indicates if p-values<0.05. The value in parenthesis indicated p-values, corrected for multiple comparisons using False Discovery Rate (q = 0.05). (PDF) [file pone.0201829.s004.pdf]

MPF	4 weeks SPF		4weeks GF		*p*-value			12 weeks SPF		12 week s GF		*p*-value		
	female	male	female	male	treatment	gender	interaction	female	male	female	male	treatment	gender	interaction
**Hippocampus**	7.59± 0.09	7.91± 0.10	7.50± 0.17	7.29± 0.16	***.013 (.048)***	.692 (.871)	.06 (.432)	7.82± 0.12	8.67± 0.24	8.05± 0.14	8.37± 0.29	.857 (.944)	***.01 (.016)***	.211 (.281)
**Corpus callosum /external capsule**	8.85± 0.17	9.35± 0.17	8.65± 0.14	8.49± 0.23	***.025 (.057)***	.453 (.871)	.156 (.432)	10.06± 0.17	11.05± 0.23	10.60± 0.21	10.95± 0.56	.43 (.821)	***.022 (.027)***	.257 (.316)
**Caudate-putamen**	7.94± 0.10	8.37± 0.15	7.93± 0.10	7.85± 0.14	.099 (.125)	.294 (.871)	.122 (.432)	8.38± 0.13	9.37± 0.23	8.68± 0.18	9.10± 0.33	.944 (.944)	***.003 (.008)***	.21 (.281)
**Anterior commissure**	7.78± 0.07	8.00± 0.09	7.69± 0.06	7.55± 0.05	***.008 (.048)***	.667 (.871)	.075 (.432)	7.94± 0.09	8.96± 0.26	8.18± 0.12	8.68± 0.15	.91 (.944)	***<0.001 (<0.001)***	.188 (.281)
**Internal capsule**	9.26± 0.22	9.49± 0.20	8.64± 0.27	8.55± 0.25	***.007 (.048)***	.799 (.871)	.568 (.606)	9.60± 0.26	10.83± 0.21	9.86± 0.39	9.92± 0.42	.315 (.821)	***.05 (.053)***	.077 (.269)
**Thalamus**	8.37± 0.09	8.69± 0.13	8.24± 0.28	8.18± 0.28	.107 (.125)	.508 (.871)	.336 (.489)	8.68± 0.18	9.94± 0.23	9.08± 0.17	9.49± 0.32	.912 (.944)	***.001 (.004)***	.082 (.269)
**Cerebellum**	8.38± 0.15	8.32± 0.13	8.04± 0.19	8.19± 0.22	.231 (.231)	.81 (.871)	.615 (.615)	8.52± 0.17	9.47± 0.28	8.49± 0.18	8.93± 0.32	.275 (.821)	***.011 (.016)***	.332 (.354)
**Hypothalamus**	7.17± 0.11	7.41± 0.10	7.03± 0.13	6.92± 0.09	.021 (.056)	.611 (.871)	.2 (.432)	7.34± 0.14	8.28± 0.16	7.66± 0.21	7.88± 0.20	.828 (.944)	***.003 (.008)***	.056 (.269)
**Central gray**	7.32± 0.10	7.44± 0.15	7.03± 0.15	6.81± 0.18	.01 (.048)	.764 (.871)	.314 (.489)	7.59± 0.16	8.59± 0.26	7.77± 0.18	8.09± 0.28	.513 (.821)	***.01 (.016)***	.167 (.281)
**Neocortex**	7.62± 0.08	7.88± 0.11	7.53± 0.08	7.49± 0.10	.05 (.086)	.365 (.871)	.216 (.432)	7.90± 0.12	8.74± 0.24	8.01± 0.14	8.35± 0.14	.482 (.821)	***.006 (.012)***	.211 (.281)
**Amygdala**	7.34± 0.10	7.58± 0.11	7.23± 0.12	7.17± 0.11	.054 (.086)	.511 (.871)	.27 (.48)	7.63± 0.12	8.60± 0.24	7.76± 0.15	8.19± 0.11	.49 (.821)	***.001 (.004)***	.184 (.281)
**Olfactory bulbs**	7.11± 0.15	7.49± 0.17	6.90± 0.20	7.05± 0.16	.109 (.125)	.183 (.871)	.56 (.606)	7.49± 0.18	8.85± 0.41	7.47± 0.24	7.66± 0.28	.077 (.821)	***.025 (.029)***	.084 (.269)
**Brainstem**	8.93± 0.13	9.16± 0.12	8.70± 0.23	8.67± 0.13	.036 (.072)	.554 (.871)	.444 (.546)	9.29± 0.13	10.51± 0.34	9.42± 0.19	9.49± 0.26	.106 (.821)	***.021 (.027)***	***.037 (.269)***
**Fimbria**	7.66± 0.14	8.05± 0.22	7.48± 0.22	7.20± 0.33	.059 (.086)	.817 (.871)	.211 (.432)	8.51± 0.15	9.48± 0.23	8.97± 0.13	9.35± 0.40	.462 (.821)	***.006 (.012)***	.206 (.281)
**Prefrontal cortex**	8.46± 0.22	8.58± 0.29	8.27± 0.51	7.80± 0.34	.17 (.181)	.621 (.871)	.41 (.546)	8.93± 0.16	10.03± 0.31	9.07± 0.37	10.43± 0.39	.375 (.821)	***<0.001 (<0.001)***	.674 (.674)
**Total brain**	8.14± 0.26	8.77± 0.28	7.81± 0.58	7.23± 0.36	***.015 (.048)***	.946 (.946)	.108 (.432)	8.58± 0.60	9.58± 0.27	9.54± 0.66	9.17± 0.67	.661 (.944)	.621 (.621)	.279 (.319)
